# Neuroinflammation in the Aging Down Syndrome Brain; Lessons from Alzheimer's Disease

**DOI:** 10.1155/2012/170276

**Published:** 2012-02-21

**Authors:** Donna M. Wilcock

**Affiliations:** Department of Physiology, Sanders-Brown Center on Aging, University of Kentucky, 800 S Limestone Street, Lexington, KY 40536, USA

## Abstract

Down syndrome (DS) is the most genetic cause of mental retardation and is caused by the triplication of chromosome 21. In addition to the disabilities caused early in life, DS is also noted as causing Alzheimer's-disease-like pathological changes in the brain, leading to 50–70% of DS patients showing dementia by 60–70 years of age. Inflammation is a complex process that has a key role to play in the pathogenesis of Alzheimer's disease. There is relatively little understood about inflammation in the DS brain and how the genetics of DS may alter this inflammatory response and change the course of disease in the DS brain. The goal of this review is to highlight our current understanding of inflammation in Alzheimer's disease and predict how inflammation may affect the pathology of the DS brain based on this information and the known genetic changes that occur due to triplication of chromosome 21.

## 1. Introduction

 Down syndrome (DS) is the most common chromosomal anomaly among live-born infants and is the most frequent genetic cause of mental retardation [1, 2], with an incidence of one per 733 live births in the United States [3]. DS is caused by a triplication of chromosome 21 (a full list of genes located on chromosome 21 can be found in [4]). Due to the extensive number of genes triplicated, there is an extremely high incidence of congenital cardiac and gastrointestinal abnormalities [5]. DS is usually detected during pregnancy through first-trimester screening tests followed up by confirmation through amniocentesis, chorionic villus sampling, or percutaneous umbilical blood sampling [6].

 Alzheimer's disease (AD) is the leading cause of dementia and is characterized clinically by a progressive loss of memory and cognition. An absolute diagnosis of AD can only occur after pathological analysis is performed on the brain tissue. There are two signature pathological lesions required for diagnosis; neuritic plaques composed of aggregated amyloid-*β* (A*β*) peptides, and neurofibrillary tangles composed of hyperphosphorylated, aggregated tau protein [7]. AD is usually considered a disease of aging, where currently 1 in 8 Americans over the age of 65 have AD yet half of those over 85 years have AD (data obtained from the Alzheimer's Association; www.alz.org).

In DS, A*β* deposits begin to accumulate in childhood and increase progressively with age [8]. There is an acceleration of this pathology between the ages of 35–45 years when other AD pathologies begin to occur, most importantly neurofibrillary tangles and inflammation [9]. Despite the certainty of developing AD-like pathologies in DS by mid-life, the onset of dementia is less certain. The consensus from a number of studies is that 50–70% of DS individuals will develop dementia by ages 60–70 years [10–13]. The reason individuals with DS develop A*β* deposits early in life is primarily due to the presence of some AD-related genes on chromosome 21, and hence these genes are triplicated in most cases of DS. Of the AD-associated genes triplicated in DS, the critical ones are amyloid precursor protein (APP) and *β*-amyloid cleavage enzyme 2 (BACE2). A*β* peptide is a cleavage product of APP. APP is a transmembrane protein and is differentially cleaved by enzymes called secretases of which there exist *α*-secretase, *β*-secretase (BACE), and *γ*-secretase. When *β*-secretase and *γ*-secretase cleave APP A*β* is a product, when *α*-secretase cleaves, this occurs in the middle of the A*β* portion and other peptides are produced.

 Inflammation is known to occur in the brains of both AD and DS patients in response to the presence of neuritic plaques and neurofibrillary tangles. This inflammation is primarily mediated by microglial cells, although other glial cells and even neurons participate in this inflammatory response. It is becoming increasingly clear in the AD field that inflammation can directly influence plaques and tangles in the same way that plaques and tangles can directly influence inflammation. The purpose of this review is to discuss the evolving understanding of neuroinflammation in AD and determine how this may relate to the pathophysiology of DS.

## 2. Neuroinflammation in Alzheimer's Disease

 Neuroinflammation is a complex process with many phenotypically varied states. The primary inflammatory cell in the brain is the microglial cell, which was first identified as a unique cell subtype by Del Rio Hortega in the 1920s. The microglial cell has been described as an ameboid-like cell that can be labeled immunocytochemically using macrophage cell surface markers [14, 15]. Other cells in the brain can contribute to the inflammatory response as well as microglia, although this contribution is considered to be significantly less than that of the microglia. Astrocytes and neurons can participate in the neuroinflammatory process as well as oligodendrocytes and vascular pericytes [16].

 The view of neuroinflammation in the brain, and in disorders of the brain, has evolved over time, and continues to evolve as our understanding of the capabilities of the system grows. While once considered “immunologically privileged,” the brain is now known to exhibit an almost complete spectrum of inflammatory responses given the correct stimuli and environment. While once considered a cytotoxic loop [17], there are now examples of harnessing the inflammatory system of the brain to ameliorate AD pathologies and improve outcomes (see further discussion later in this section).

 In AD, microglia expressing some classic activation markers such as MHC-II (associated with antigen presentation), CD68 (a lysosomal protein), and CD36 (a class B scavenger receptor) are highly localized to the area immediately surrounding an amyloid plaque or neurofibrillary tangle [18]. While this led some to hypothesize that this reaction was contributing to the toxicity of these pathologies, others suggested that the microglia may be performing a beneficial function in removing the abnormal protein deposits from the brain. As yet, there is no consensus, and it is likely that both phenomena are occurring to differing degrees. To better understand these processes, researchers turned to the assessment of cytokines to determine the function(s) of these microglial cells.

 In AD, many cytokines have been found to be altered. Among those, the most common are IL-1*β*, IL-6, TNF*α*, and TGF*β*. IL-1*β* was first shown by Griffin et al. in 1995 to be associated with the development of neuritic amyloid plaques from diffused deposits using human postmortem tissue [19]. Later, Griffin et al. expanded their findings to develop a “cytokine cycle” hypothesis that suggested the IL-1*β* production in response to amyloid deposits initiated a series of events including increased APP production and processing by neurons, recruitment of astrocytes, and activation of these astrocytes leading to signaling in the microglia inducing yet further IL-1*β* [20]. IL-1*β* induces S100*β* production in astrocytes [21], which is a cytokine that promotes neurite growth [22]. Most recently, serum IL-1*β* has been found to be elevated in cases of mild cognitive impairment (MCI) that has a higher risk for conversion to dementia, possibly indicating that serum IL-1*β* may be useful for identifying those MCI patients at risk for converting to AD [23]. Also, there are genome-wide association studies (GWASs) that have identified IL-1*β* polymorphisms associated with AD (reviewed in [24]). It will require further studies and analyses to determine whether these polymorphisms, are, in fact, associated with AD risk. However, in contrast to the negative data presented with respect to IL-1*β*, there is more recent data showing that IL-1*β* overexpression in the hippocampus of transgenic mice results in amelioration of amyloid pathology. IL-1*β* was increased specifically in a single hippocampus of an APP/PS1 transgenic mouse by genetic means and this hippocampus showed a 50% reduction in plaque load [25].

IL-6 is another cytokine that mediates immune responses and inflammatory reactions [26]. While microglia are the main source of IL-6 in the CNS, astrocytes, neurons, and endothelial cells are all capable of producing the cytokine [27–29]. In AD, brain tissue IL-6 has been shown to be elevated in pathologically relevant regions [30]. While much of the focus on IL-6 has been on its destructive effects such as induction of acute-phase proteins, increasing vascular permeability, activation of lymphocytes, and antibody synthesis (reviewed in [31]), there are some positive effects of IL-6 that may play a role in AD. This includes enhancing neuronal survival [32–34] and suppressing demyelination in a model of multiple sclerosis [35]. Moreover, in a mouse model of amyloid deposition, Chakrabarty et al. showed that overexpression of IL-6 enhanced microglial phagocytosis of amyloid deposits and, therefore, ameliorated amyloid burden [36].

 TNF*α* is another cytokine that has been shown to have both beneficial and detrimental effects in the CNS. It acts as a highly potent proinflammatory and cytotoxic molecule in conditions of the CNS [37–40]. In contrast, TNF*α* has been shown to have trophic effects on hippocampal neurons [41] and provide protection from free-radical damage in primary neurons [42]. It is thought that the source of such dichotomous effects is the receptor subtype through which the TNF*α* is acting. There are two primary receptors for TNF*α* in the CNS; TNF*α* receptor 1 (TNFR1) and TNF*α* receptor 2 (TNFR2) [43]. TNFR1 mediates neuronal death via the TNF-receptor-associated death domain protein and caspase-8-activated apoptosis [44, 45]. TNFR2 is thought to mediate the beneficial, prosurvival action of TNF*α* through the nuclear factor-*κ*B- (NF*κ*B-) mediated antiapoptotic pathway [46]. This is likely an oversimplified view of the actions of TNF*α* through its receptors and there have been many subtleties of these systems described in the literature. In AD, it has been shown that expression of TNFR1 is elevated in the brain while levels of TNFR2 are decreased [44]. In addition, clinical trials are ongoing for the treatment of AD with etanercept, a fusion protein combining TNFR2 and the Fc portion of IgG used to treat Crohn's disease and arthritis as well as other autoimmune disorders [47]. Etanercept acts as a decoy receptor for TNF, reducing the effects of TNF at the biologically active receptors. Preliminary studies showed that perispinal delivery of etanercept in a small number of AD patients improved cognition [48]. In addition, thalidomide is also currently in clinical trials for AD based on its anti-TNF*α* effects. In transgenic mice, thalidomide has been shown to improve learning and memory [49].

 Finally, TGF*β* is a growth factor that has been shown to play a prominent role in tissue development, homeostasis, and repair [50]. Unlike the cytokines discussed to this point, TGF*β* is associated mostly with repair mechanisms and is not known for its damaging or cytotoxic actions in the CNS. Instead, it is mostly associated with the formation of a glial scar [51] and upregulation of extracellular matrix proteins [52–54]. In AD, TGF*β* levels are increased in the brain [55] but decreased in serum [56]. In APP transgenic mice, overproduction of TGF*β* by astrocytes results in lower parenchymal amyloid deposits but increased deposition of amyloid in the cerebrovasculature [57]. Most recently, Tesseur et al. have shown that deficiencies exist in TGF*β* signaling in the human AD brain, and these deficiencies can lead to enhanced AD pathology and associated neurodegeneration [58].

 There is a rapidly growing interest in better characterizing the inflammatory state in the brain, and especially in AD. A paper by Colton et al. in 2006 described “classical activation” and “alternative activation” of microglia in the brain [59]. Classical activation was used to describe the Th1 cytokines such as IFN*γ*, IL-1*β*, TNF*α*, and IL-6. Alternative activation was used to describe a state associated with anti-inflammatory, repair, and wound healing effects mediated by IL-10, TGF*β*, IL-4, IL-13, arginase 1 (AG1), and tissue remodeling factors Found in Inflammatory Zone 1 (FIZZ1) and chitinase 3-like 3 (YM1). This paper showed that cultured microglial cells, transgenic mouse models of AD, and postmortem tissue from human AD brains all showed expression of both classical and alternative activation markers. Most interesting was that alternative activation markers were expressed to the same degree, sometimes more than the classical activation markers commonly associated with an inflammatory response.

**Figure 1:**
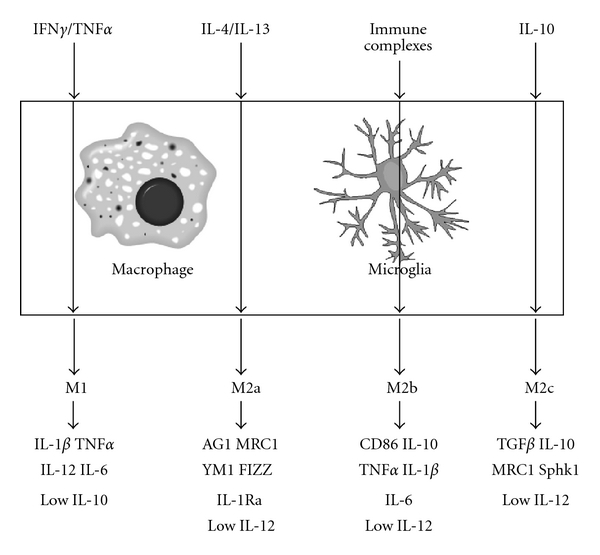
Schematic showing the four distinct states of inflammation possible in response to a stimuli in microglial/macrophage cells.

 We have now expanded on the concept of multiple activation states to include a full spectrum of macrophage responses. Shown in Figure 1 are the four distinct inflammatory states we are currently studying in the brain. These states are well characterized in the peripheral macrophage literature (reviewed in [60, 61]). The M1 response is stimulated by IFN*γ* and/or TNF*α* and is characterized by traditional inflammatory cytokines IL-1*β*, IL-6, and IL-12. Broadly, the M2 response represents the alternative activation state described by Colton et al. We can further categorize this state into M2a, M2b, and M2c. Each subtype of M2 response has distinct stimuli and responses. IL-4 and/or IL-13 initiate an M2a response that is characterized by tissue remodeling factors FIZZ and YM1 as well as AG1 and mannose receptor C1 (MRC1). Immune complexes stimulate an M2b response, which is a specific response that has components of both M1 and M2a states. Finally, IL-10 stimulates an M2c response, which is sometimes called an acquired deactivation state. The M2c response is characterized by a series of markers that actively antagonize M1 signaling pathways. By categorizing the inflammatory response into these distinct types where each stimuli and marker is established, we can better understand what role(s) each state plays in AD progression and therapy.

 Drug development for the treatment of AD has recently been harnessing the inflammatory component of the disease for treatment. The most interesting approach is immunotherapy for AD. First demonstrated in 1999 [62], immunotherapy uses either an active vaccination approach or passive immunization to introduce anti-A*β* antibodies in patients (reviewed in [63]). These anti-A*β* antibodies then result in reductions in A*β* in the brain and ultimately, at least in transgenic mouse models, improvements in learning and memory [64, 65]. Injection of anti-A*β* antibodies directly into the brains of transgenic mouse models showed a dependence of amyloid removal on microglial activation [66, 67]. Later studies systemically administering anti-A*β* antibodies also showed a transient activation of microglia [68] and a reduced efficacy when the antibody was deglycosylated; a process that renders the IgG molecule incapable of interacting with effector cells such as microglia [69].

 Another approach that targets the inflammatory response is the administration of nonsteroidal anti-inflammatory drugs (NSAIDs). NSAIDs showed great promise in retrospective epidemiological studies finding significant protection from AD with long-term NSAID use [70]. However, a prospective clinical trial performed by NIA/NIH, called the ADAPT trial, failed to show any significant benefit [71]. The NSAID story was furthermore clouded because some NSAIDs also possessed *γ*-secretase modifying properties that shifted APP cleavage to promote A*β*38 production, as opposed to A*β*40 or A*β*42 [72]. The NSAIDs found to have this activity were not included in the NIA/NIH trial. However, it was recently found that a subset of patients in the ADAPT trial did, in fact, benefit from NSAID use. Naproxen attenuated cognitive decline in a subgroup of AD patients termed “slow decliners,” whereas cognitive decline was accelerated in those termed “fast decliners” [73]. It is unclear why this would be the case, however, it is possible that different inflammatory states may exist in these different AD cases; some benefit from NSAIDs while some do not. Future studies will examine whether this is, indeed, the case.

## 3. Neuroinflammation in Down's Syndrome

 While many of the pathways of inflammation described for AD will be directly relevant to DS, there are some critical inflammatory genes on chromosome 21 that will be triplicated in DS and may, therefore, influence the inflammatory state of the DS brain. We will discuss those factors in this review and take our current knowledge of inflammatory states in other neurological disorders to predict how these may be playing a role in DS.  Table 1 shows the inflammatory-associated genes that are found on chromosome 21 and are triplicated in most DS patients. We will discuss each of these factors and their impact on the inflammatory balance of the brain. 

CXADR is a gene encoding for a protein called coxsackie virus and adenovirus receptor (herein abbreviated CXADR). CXADR has a dual function as a viral receptor and an adhesion molecule associated with tight junctions. It is highly expressed in brain as well as systemic secretory organs such as the pancreas, testis, and small intestine [74]. In the heart, CXADR is increased in models of myocardial inflammation and cardiac injury in the absence of viral infection suggesting that there is an innate role of this protein in the inflammatory response [75]. Recently, it was shown that CXADR can induce stress-activated mitogen-activated protein kinase (MAPK) pathways in the heart leading to increased production of IFN*γ*, IL-12, IL-1*β*, TNF*α*, and IL-6 [76]. One can predict then that increased expression of CXADR in Down's syndrome may contribute to an overactivated M1 inflammatory response, since all of these inflammatory cytokines induced by CXADR are associated with an M1 response. In addition, CXADR has a significant role in tight junction function where, in endothelial cells, it facilitates transendothelial migration of neutrophils [77]. If CXADR expression is altered on the endothelial cells of the cerebrovasculature in DS patients, then there may be altered infiltration of peripheral inflammatory cells into the brain influencing the inflammatory response.

 Two members of the ADAMTS (a disintegrin and metalloproteinase with thrombospondin motif) family are located on chromosome 21 and, therefore, subject to triplication in DS, ADAMTS1, and ADAMTS5. ADAMTS1 contains a signal peptide in the N-terminal region indicating it is secreted [78]. It acts as a proteinase degrading extracellular matrix proteoglycans such as aggrecan and versican [79]. ADAMTS5 is also a proteinase and shares the same substrates as ADAMTS1 [80]. Both ADAMTS1 and ADAMTS5 can be induced by IL-1*β*, indicating a dependence on an inflammatory response [81, 82]. It has been shown in DS that ADAMTS1 is five-fold overexpressed at the protein level, while ADAMTS5 was not significantly increased by Western blot measurements [83]. Given the induction by inflammatory cytokine IL-1*β*, one could hypothesize that the triplication of these proteinases would lead to exacerbated degradation of extracellular matrix proteins in response to an inflammatory insult. In addition, Griffin et al. showed that DS brain has greater IL-1*β* immunoreactivity indicating that there is more IL-1*β* present in the DS brain to stimulate the ADAMTSs [84]. 

 T-cell lymphoma invasion and metastasis 1 (TIAM1) is a guanine nucleotide exchange factor for Rac1 [85] and, therefore, contributes to the activation of Rac1, which is necessary for the activation of NADPH oxidase [86]. Most recently, Tiam1 was found to be a critical regulatory factor in cytokine-induced induction of NADPH oxidase, more specifically, induction by IL-1*β* [87]. While these data used pancreatic *β*-cells, one could predict that overexpression of Tiam1 in the DS brain could lead to increased oxidative stress in response to an inflammatory insult that involves IL-1*β*. Indeed, it has been shown that Tiam1 protein expression is increased in fetal DS brain compared to control fetal brain [88].

 Superoxide dismutase 1 (SOD1) binds copper and zinc and is a potent endogenous antioxidant. The enzyme is a soluble cytoplasmic and mitochonidral interspace protein that converts superoxide radicals to molecular oxygen and hydrogen peroxide [89]. Mutations in the SOD1 gene are commonly associated with genetic susceptibility to anterolateral sclerosis (ALS) [90]. While the hypothesis for the role of these mutations centered on the potential loss of function, and, therefore, increased oxidative stress, there has been increasing evidence to discount this hypothesis including the lack of ALS symptoms or pathology in SOD1 knockout mice [91]. It is unclear what the consequence is of overexpression of nonmutant SOD1 as would occur in DS. In a model of retinitis pigmentosa, it was found that loss of SOD1 worsened the outcomes. However, when SOD1 was overexpressed in this model, the levels of oxidative damage were actually worse. The authors found that in the absence of a peroxide-detoxifying enzyme in the same cellular compartment, overexpression of SOD1 actually causes more oxidative stress [92]. It could be suggested that the same may be the case in DS if the triplication of SOD1 results in overexpression of the protein in the absence of an increased level of peroxide-detoxifying enzyme.

 Interferon receptors IFNAR1, IFNAR2, and IFNGR2 are all located on chromosome 21 and are, therefore, all subject to triplication in most cases of DS. IFNAR1 and IFNAR2 both respond to IFN*α*, IFN*β*, or IFN*ο* and, upon ligand binding, activate the JAK/STAT signaling pathway leading to induction of proinflammatory gene expression such as IL-1*β*, TNF*α*, and IL-6. IFNGR2 uses the same signaling pathway but responds to IFN*γ* specifically. A mouse model for the study of DS, the trisomy 16 mouse, includes triplication of IFNGR2 and IFNAR2. These mice develop significant pathology *in utero* and rarely survive to birth. Studies in these mice have shown that anti-IFN IgG treatment of fetuses improves the mouse phenotype suggesting the triplication of the IFN receptors significantly contributes to the severe pathology present in these mice [93]. The same group later showed that introducing a partial knockout of the IFNAR2 and IFNGR2 can improve growth and viability of cultured neurons derived from the trisomy 16 mouse fetuses [94]. Since these genes are triplicated in DS, it is likely that there is a hyperresponsiveness to IFN in the DS patient that may lead to an increased inflammatory response, both in the brain and systemically.

 Receptor-interacting serine-threonine kinase 4 (RIPK4) is a protein kinase involved in multiple cell signaling pathways. One of these pathways is the signaling pathway for the activation of NFkB [95]. In addition, RIPK4 is involved in the signaling cascade of the TNF*α* receptor TNFR1 [96]. It is important to note that the TNFR1 is most heavily implicated with the toxic effects of TNF*α* and it could be predicted that overexpression of RIPK4 may increase responsiveness of TNFR1 to TNF*α* exacerbating the effects of TNFR1. At this time, however, this is purely speculative.

 Cytathione beta synthase (CBS) is a cytosolic enzyme that catalyzes the desulfhydration of cysteine-producing hydrogen sulfide (H_2_S). H_2_S is now recognized as an atypical cellular messenger that has many normal physiological functions [97]. CBS binds NO or CO in its heme pocket and this binding modulates the activity of the enzyme [98]. H_2_S is a complicated signaling molecule with an apparent bimodal action on inflammation, where low levels appear to be anti-inflammatory, yet high levels may exacerbate inflammation in some instances. There are several extensive reviews on H_2_S signaling that discuss this phenomenon in great detail (see [99, 100]). It remains unclear how the overexpression of CBS in DS influences the DS pathology and whether the amount of H_2_S produced in DS patients is of the anti-inflammatory or proinflammatory concentrations.

 S100*β* is a protein localized primarily to the brain where it is expressed by astrocytes. It is secreted by astrocytes in response to IL-1*β* and cyclic-AMP [101]. S100*β* is another inflammatory mediator with dichotomous actions. At low concentrations, it appears to enhance survival of neurons [102] and stimulate neurite outgrowth [22]. In contrast, high concentrations of S100*β* increases cell death [103] and causes apoptosis [104]. It has been shown in DS brains that S100 is greatly increased compared to control brain. The concentrations would place the levels of S100*β* in the toxic category, suggesting that the overexpression of S100*β* in DS brain plays a negative role in the aging pathology [84].

 Protein arginine methytranferase 2 (PRMT2) is an enzyme that catalyzes the methylation of arginine. It has been shown that arginine methylation is a means of regulation of the JAK-STAT signaling pathway, which is key for many inflammatory processes including IFN*γ*, IFN*α*, and IL-6 [105]. In addition, natural degradation of proteins containing methylated arginine results in the production of asymmetric dimethylarginine (ADMA) [106]. ADMA is an endogenous inhibitor of nitric oxide synthase (NOS), a key player in normal cell signaling and inflammation [107]. It is unclear whether the triplication of PRMT2 results in significant changes in ADMA concentrations in the brain, however, DS patients with pulmonary hypertension do show increased ADMA concentrations compared to non-DS patients with pulmonary hypertension [108]. If this were also true for the brain, one could predict that there would be decreased production of NO and increased activation of the JAK-STAT pathway, both factors could influence the inflammatory state of the brain. 

## 4. Inflammation Hypothesis and Future Directions

**Figure 2:**
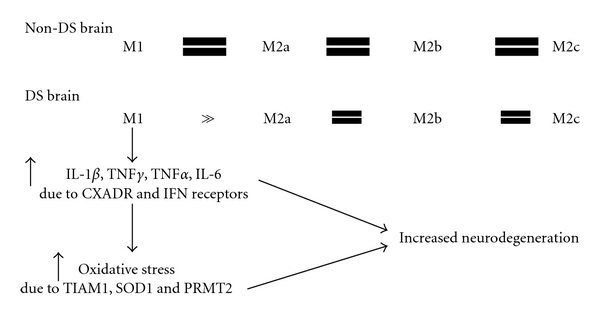
Schematic illustrating our hypothesis for the role of inflammation in Down syndrome.

 We hypothesize that the triplication of chromosome 21 as occurs in DS will result in a greatly exacerbated M1 inflammatory response. The basis for this hypothesis is the range of genes that are found on chromosome 21 and, therefore, triplicated. We have discussed each of the genes that are relevant to inflammation above and have summarized what these may mean to inflammation in Figure 2. Since most of the genes are primarily associated with the M1 inflammatory response, we predict that this is the main state that will be enhanced in the DS brain. Triplication of the major interferon receptors IFNAR1, IFNAR2, and IFNGR2 means that there will be enhanced interferon signaling. In turn, this enhanced signaling will increase production of M1 markers IL-1*β*, TNF*α*, and IL-6. While these components are known to result in oxidative stress, the triplication of TIAM1, SOD1, and PRMT2 will greatly exacerbate this oxidative stress. TIAM1 enhances oxidation by inducing NADPH oxidase, SOD1 at high concentrations has been shown to enhance oxidation, and PRMT2 inhibits nitric oxide production, which acts as an antioxidant in the brain at physiologic concentrations. All of these factors will combine to enhance neurodegeneration in the DS brain in response to primary pathologies such as amyloid plaques and neurofibrillary tangles.

 In considering inflammation in DS, there is a relative lack of data relative to other disorders. While AD provides us with significant background information on the role of inflammation in the disease, it is clear that the condition of DS, and the triplication of so many inflammatory-associated genes, creates a unique inflammatory environment worthy for further study. The data obtained through the study of inflammation in DS will be essential to further not only the study of DS but also, in turn, the normal inflammatory pathways in neurodegenerative disorders.

## Figures and Tables

**Table 1:** A summary of the inflammation-related genes located on chromosome 21.

Gene	Protein	Function	Ref
CXADR	Coxsackie virus and adenovirus receptor	Activation of JNK and p38-MAPK pathways leading to production of M1 cytokines.	[76]
ADAMTS1	ADAM metalloproteinase with thrombospondin type 1 motif, 1	Secreted protease known to be induced by IL-1*β*	[81]
ADAMTS5	ADAM metalloproteinase with thrombospondin type 1 motif, 5	Secreted protease known to be induced by IL-1*β* and TGF*β*.	[82]
TIAM1	T-cell lymphoma invasion and metastasis 1	Necessary for cytokine-mediated generation of oxidative species through NADPH oxidase.	[87]
SOD1	Superoxide dismuatose 1	Scavenges superoxide radicals producing H_2_O_2_ and O_2_.	[109]
IFNAR2	Interferon (alpha, beta, and omega) receptor 2	Activates JAK/STAT-mediated pathway in response to IFN*α*/*β*.	[110]
IFNAR1	Interferon (alpha, beta, and omega) receptor 1	Activates JAK/STAT-mediated pathway in response to IFN*α*/*β*.	[110]
IFNGR2	Interferon gamma receptor 2	Activates JAK/STAT-mediated pathway in response to IFN*γ*.	[111]
RIPK4	Receptor-interacting serine-threonine kinase 4	Necessary for signaling through TNFR1	[96]
CBS	Cystathione-beta-synthase	Production of hydrogen sulfide (H2S); a regulator of inflammation	[112]
S100B	S100 calcium binding protein B	Constitutive expression by astrocytes, released in response to TNF*α*	[113]
PRMT2	Protein arginine methyltransferase 2	Blocks the actions of NF*κ*B in the nucleus	[114]
